# Progress and Innovative Combination Therapies in Trop-2-Targeted ADCs

**DOI:** 10.3390/ph17050652

**Published:** 2024-05-17

**Authors:** Yizhi Jiang, Haiting Zhou, Junxia Liu, Wentao Ha, Xiaohui Xia, Jiahao Li, Tengfei Chao, Huihua Xiong

**Affiliations:** Department of Oncology, Tongji Hospital, Tongji Medical College, Huazhong University of Science and Technology, Wuhan 430030, China; m202376699@hust.edu.cn (Y.J.); doctorzhouht@163.com (H.Z.); d202282220@hust.edu.cn (J.L.); d202382340@hust.edu.cn (W.H.); m202176434@hust.edu.cn (X.X.); ljh362531@163.com (J.L.)

**Keywords:** Trop-2, antibody–drug conjugates, drug delivery systems, extracellular vesicles, personalized therapy, biomarker identification, combination therapy

## Abstract

Precise targeting has become the main direction of anti-cancer drug development. Trophoblast cell surface antigen 2 (Trop-2) is highly expressed in different solid tumors but rarely in normal tissues, rendering it an attractive target. Trop-2-targeted antibody-drug conjugates (ADCs) have displayed promising efficacy in treating diverse solid tumors, especially breast cancer and urothelial carcinoma. However, their clinical application is still limited by insufficient efficacy, excessive toxicity, and the lack of biological markers related to effectiveness. This review summarizes the clinical trials and combination therapy strategies for Trop-2-targeted ADCs, discusses the current challenges, and provides new insights for future advancements.

## 1. Introduction

Cancer remains a crucial driver of worldwide mortality, with an estimated 20 million new diagnoses and 10 million fatalities in 2022 [1]. For decades, cytotoxic agent-based chemotherapy has been the predominant treatment modality for a diverse range of cancers [2]. However, a majority of these chemotherapy agents have a narrow therapeutic window, attributed to substantial off-target tissue exposure and consequent severe adverse effects [3]. Therefore, targeted therapy has emerged as a promising strategy for cancer treatment, attributed to its demonstrated efficacy and favorable safety profile [4]. Antibody–drug conjugates (ADCs), as a type of targeted therapy, accurately pinpoint tumor cells by connecting specific monoclonal antibodies with powerful chemotherapy drugs through stable linkers [5]. In comparison to traditional chemotherapy, ADCs offer increased tumor selectivity, enhanced efficacy, and reduced toxic side effects and have garnered significant attention in recent research [6]. Reaching a growing array of targets and indications, ADCs are leading the path towards a fresh era in precise cancer therapy [7].

**Figure 1:**
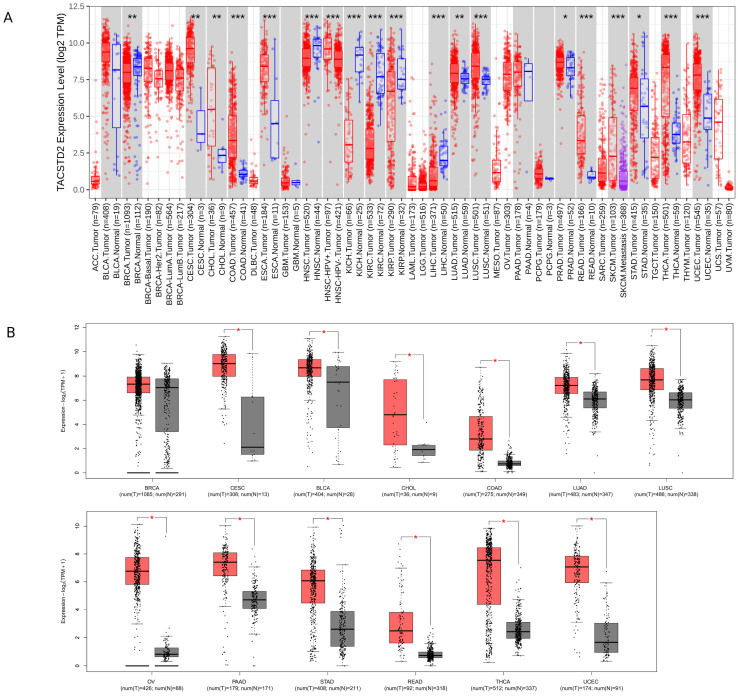
(**A**) The expression of TACSTD2 in various tumor tissues (red) and adjacent normal tissues (blue) was analyzed via the TIMER2.0 database (http://timer.cistrome.org/ (accessed on 10 May 2024)) (* *p*-value < 0.05; ** *p*-value < 0.01; *** *p*-value < 0.001). (**B**) Tumors with high Trop-2 expression (red: tumor tissue, grey: normal tissue; * *p* < 0.05) that could potentially benefit from Trop-2-targeted ADC therapeutics (GEPIA database http://gepia.cancer-pku.cn (accessed on 10 May 2024)).

Trophoblast cell surface antigen 2 (Trop-2), a type I transmembrane glycoprotein, is overexpressed in various epithelium-derived tumors (Figure 1) and is strongly associated with malignant behavior. Consequently, it has been identified as an appealing target for cancer therapy [8]. The approval of the first Trop-2-targeted ADC, IMMU-132, in April 2020 has ushered in new possibilities in cancer treatment [9]. The success of IMMU-132 has raised the hope for new treatments for patients suffering from refractory cancer. This review provides a comprehensive overview of drug development, combination therapy, and potential enhancement strategies in Trop-2-targeted ADCs.

## 2. Trop-2

Trophoblast cell surface antigen 2 (Trop-2) is encoded by the TACSTD2 gene found on chromosome 1p32 [10,11]. This 36-kDa type I transmembrane glycoprotein is made up of 323 amino acids and consists of various sections: a hydrophobic signal peptide (amino acids 1 to 26), an extracellular domain (amino acids 27 to 274), a transmembrane domain (amino acids 275 to 297), and a cytoplasmic tail (amino acids 298 to 323, which interact with the antibody). The extracellular domain has four N-glycosylation locations and features sequences found in the epidermal growth factor-like (EGF-like) region, with a rich cysteine area, a thyroglobulin-1 area, and a cysteine-sparse area [12]. Trop-2 is involved in regulating the cell cycle and is linked to proliferation and differentiation. Its mechanisms of action involve increasing Ki-67 expression, promoting the expression of cyclins D and E, mobilizing intracellular calcium stores to stimulate cell proliferation, and directly activating the MAPK signaling pathway and downstream AP-1 to foster cell differentiation and proliferation [13]. TROP2 overexpression not only supports tumorigenic cell proliferation, differentiation, and metastasis through pathways like JAK/STAT, Bax, Bcl-2, ERK, and Akt but also enhances PARP1 expression, leading to increased DNA replication and damage accumulation. Furthermore, the activation of Trop-2’s intracellular domain (ICD) can drive tumor cell proliferation; induce epithelial–mesenchymal transition (EMT); and boost tumor cell migration, invasion, and anti-apoptotic capabilities, ultimately hastening tumor progression [14].

## 3. Trop-2-Targeted ADC

### 3.1. Development of Trop-2-Targeted ADCs

**Figure 2:**
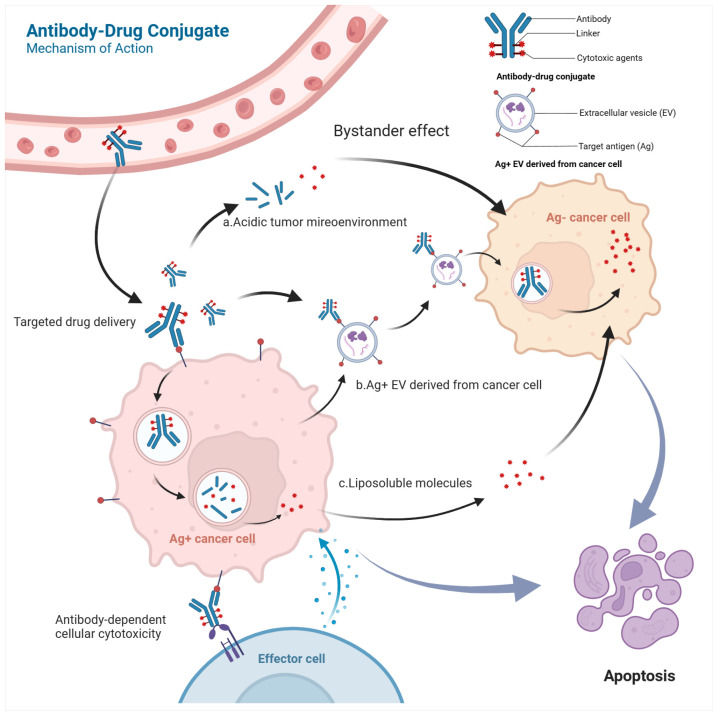
Mechanism of action of ADCs: (a) PH-sensitive linkers enable ADCs to release cytotoxic drugs in the acidic tumor microenvironment, where tumor cells generate a lot of lactic acid through glycolytic metabolism (known as the Warburg effect), allowing certain ADCs to exert anti-tumor effects without complete integration of the conjugates. (b) ADCs bind to extracellular vesicles (EVs) that are released by cancer cells expressing specific target proteins. These EVs serve as carriers, transporting the ADCs to nearby and distant cancer cells, resulting in anti-cancer effects and potentially altering the adverse effects associated with ADC treatment. (c) Liposoluble cytotoxic drugs can permeate the phospholipid bilayer, allowing them to be released from Ag+ cancer cells and impacting nearby Ag− cancer cells, ultimately playing an anti-tumor role.

Antibody–drug conjugates (ADCs) consist of human monoclonal antibodies (mAbs), linkers, and cytotoxic agents [7]. The mAbs specifically target antigens on the cell membrane of the desired cells and undergo internalization via endocytosis (Figure 2). Upon intracellular entry, ADCs transit to early endosomes and lysosomes, where they degrade under acidic conditions or with enzymatic activity. This breakdown releases cytotoxic drugs, which subsequently bind to DNA or microtubule proteins, inducing cell cycle arrest and eventual apoptosis [15]. The advancement of Trop-2-targeted ADCs has seen rapid progress in recent years, with multiple compounds currently undergoing clinical investigation [16,17,18,19].

#### 3.1.1. Sacituzumab Govitecan

Sacituzumab govitecan (SG/IMMU-132) is the groundbreaking ADC that targets Trop-2. It comprises the humanized IgG1 monoclonal antibody hRS7 (KD = 0.3 nM) attached to SN-38, a topoisomerase inhibitor, via the cleavable linker CL2A. This innovative design targets patients with breast cancer and urothelial cancer [20,21,22]. The pH-sensitive CL2A linker facilitates the intracellular and extracellular release of the active payload, promoting a bystander effect that enhances the drug’s efficacy [23]. Moreover, SG has shown promising results in advanced and metastatic non-small-cell carcinoma (NSCLC) and invasive bladder cancer [24,25,26]. Its successful market introduction has set a precedent for Trop-2-targeted cancer therapy. The final study results of the phase III clinical trial TROPiCS-02, released in August 2023, demonstrated the efficacy of SG in patients with hormone receptor-positive/human epidermal growth factor receptor-2-negative metastatic breast cancer (HR+/HER2-mBC), leading to a significant improvement in their progression-free survival [27]. The data from TROPiCS-02 have resulted in the inclusion of SG as a category I option in the 2023 NCCN Guidelines for Breast Cancer and its recommendation as a second-line treatment for HR+/HER2− breast cancer patients who have previously received endocrine therapy (ET) and CDK4/6 inhibitors [21].

#### 3.1.2. Datopotamab Deruxtecan

Datopotamab deruxtecan (Dato-DXd), the second ADC drug developed using the DXd technology platform, exhibits a potent bystander effect in the treatment of breast cancer and non-small-cell lung cancer [28]. MAAP-9001a, a humanized IgG1 antibody with low affinity (KD = 27 nM) to Trop-2 targets, was intended to broaden the drug’s therapeutic window, balancing efficacy and safety. The results from the TROPION-Lung01 study suggest that Dato-DXd monotherapy improves survival outcomes in locally advanced/metastatic NSCLC patients, regardless of actionable genomic alterations (AGA) status. Specifically, non-squamous cell carcinoma NSCLC patients demonstrate a particularly strong response to Dato-DXd treatment [29]. The phase II clinical trial TROPION-Lung05 further demonstrates the substantial anti-tumor activity and manageable safety profile of Dato-DXd in AGA patients with advanced/metastatic NSCLC who have undergone multiple lines of therapy [30,31]. Additionally, Dato-DXd displays promising efficacy and safety in the triple-negative breast cancer (TNBC) cohort and the HR+/HER2− cohort of TROPION-PanTumor01 [32,33]. Further clinical studies are underway to explore the potential application of Dato-DXd in HER2-negative breast cancer [34,35].

#### 3.1.3. SKB264

SKB264 is an innovative ADC that targets Trop-2, utilizing the same antibody component as IMMU-132. The payload consists of a derivative of belotecan, a potent topoisomerase I inhibitor known as KL610023 (T030), linked via an enzymatically cleavable linker to enhance stability and reduce toxic side effects [36,37]. Initial clinical results show that SKB264 effectively treats advanced TNBC and other Trop-2-expressing solid tumors, with manageable side effects. Ongoing clinical trials are investigating the safety, tolerability, and objective response rate of SKB264 as a monotherapy or in combination with other treatments for advanced and metastatic NSCLC.

#### 3.1.4. SHR-A1921

SHR-A1921 comprises a unique topoisomerase I inhibitor, SHR9265, connected to a proprietary IgG1 monoclonal antibody with a cleavable linker [19]. This ADC demonstrates superior binding affinity to human and cynomolgus monkey Trop-2 compared to existing ADCs targeting Trop-2, resulting in enhanced therapeutic effectiveness. The carefully designed spatial arrangement of the payload minimizes non-specific cleavage, ensuring sustained drug activity in vivo. Additionally, the high lipophilicity of the payload allows it to overcome tumor heterogeneity and resistance, producing a bystander effect. In clinical comparisons, SHR-A1921 exhibits a significantly longer half-life than SKB264, suggesting the potential for less frequent dosing and reduced patient burden. Overall, with its deep tissue penetration, optimized drug-to-antibody ratio, and proven stability and efficacy in preclinical trials, SHR-A1921 has emerged as a strong candidate in Trop-2-targeted ADC therapy.

As additional clinical trial data are gathered, it is anticipated that more Trop-2-targeted ADC medications will receive approval, offering novel treatment avenues for individuals battling cancer.

### 3.2. Drug Combination Therapy

The potential of ADCs in cancer treatment is hindered by the heterogeneity and individual variability of malignant tumors, making it difficult to achieve optimal therapeutic efficacy using a single agent or therapy. Consequently, the focus has shifted towards exploring combination therapies that involve the use of multiple drugs. This approach is seen as a promising strategy in anti-tumor drug research, as it allows for the targeting of various pathways and mechanisms of cancer cell growth and proliferation. Several clinical trials to investigate potential drug combination strategies are currently in progress (refer to Table 1 for details).

#### 3.2.1. Combination with Chemotherapy

Combining ADCs with chemotherapeutic agents enhances the anti-tumor effect and reduces drug resistance. However, it is crucial to consider the timing of drug administration and potential drug toxicity when using combination regimens. Global clinical trials are currently in progress to assess the effectiveness of Dato-DXd in combination with platinum-based chemotherapy in different settings. For example, the TROPION-Lung02 study assessed the Trop-2-targeted ADC in combination with immunotherapy (pembrolizumab) ± platinum-based chemotherapy in advanced NSCLC. This study demonstrated that the combination of Dato-DXd with immune checkpoint inhibitors and chemotherapy was well-tolerated and had significant anti-tumor activity in patients with advanced NSCLC. In terms of safety, 61% of patients experienced grade ≥ 3 treatment-emergent adverse events (TEAEs), with common occurrences including nausea, anemia, malaise, and stomatitis [38]. Ongoing studies such as TROPION-Lung07 and AVANZAR are further exploring the potential of Trop-2-targeted ADC combination therapy. The TROPION-Lung07 study is explicitly investigating the efficacy and safety of Dato-DXd + immune checkpoint inhibitor ± chemotherapy in patients with advanced/metastatic NSCLC with PD-L1 < 50% [39]. On the other hand, the AVANZAR study will assess the efficacy and safety of Dato-DXd + dolvarizumab + carboplatin as a first-line treatment for advanced/metastatic NSCLC [40].

#### 3.2.2. Combination with Molecular-Targeted Drugs

Molecular-targeted drugs encompass two main categories: those that target tumor cells directly and those that target the tumor growth microenvironment. The molecular-targeted drugs currently used in conjunction with Trop-2-targeted ADCs include PARP inhibitors, ATR inhibitors, and CDK4/6 inhibitors, among others. The combination of molecular-targeted drugs and ADCs shows significant promise in enhancing anti-tumor effects, reducing drug toxicity, and overcoming ADC resistance. The phase Ib SEASTA study demonstrated that the combination of rucaparib and SG exhibited potent anti-tumor activity in patients with advanced solid tumors, including those who had previously received PARP inhibitors and tumors with or without HRR gene mutations [41]. Another phase I study indicated that the ATR inhibitor berzosertib combined with SG showed a favorable safety profile compared to traditional chemotherapy without dose limitations. This combination also led to a lower occurrence of hematological toxicity than the combination of berzosertib and the conventional TOP1 inhibitor topotecan [42]. A phase II trial is currently underway to assess the safety and effectiveness of SG in combination with a CDK4/6 inhibitor (trilaciclib) in patients with TNBC. The initial data from the first 18 patients revealed a significant reduction in multiple associated adverse events compared to the results obtained using SG as a single agent [43]. Furthermore, the use of Ceralasertib (ATRi) in combination with Dato-DXd demonstrated the ability to re-sensitize Dato-DXD-resistant NCI-N87 cells in vitro and in vivo, potentially offering new strategies for combating ADC resistance and identifying biological markers of drug sensitivity [44].

Tumor progression requires the induction of tumor angiogenesis, with pro-angiogenic factors such as VEGF and PDGF mainly produced by tumor and stromal cells in the tumor microenvironment. Increased vascularization contributes to tumor recurrence, progression, invasion, and metastasis. The combination of antiangiogenic drugs with ADCs has the potential to target both tumor cells and the microenvironment, with current clinical trials exploring this approach in various cancer types. This strategic combination is expected to improve therapeutic outcomes, reduce the development of drug resistance, and potentially minimize side effects compared to monotherapy, offering promising options for cancer patients [45].

#### 3.2.3. Combination with Immunotherapy

In clinical trials, immune checkpoint inhibitors (CPIs) such as CTLA-4 antibodies, PD-1 antibodies, and PD-L1 antibodies have been utilized in conjunction with Trop-2-targeted ADCs [46,47,48,49]. Research indicates that ADCs can stimulate the infiltration of CD8+ T-cells into tumor tissue, with these cells being the primary effector population driving anti-tumor responses in many cancer immunotherapy settings. ADCs promote anti-tumor immunity by activating and maturing dendritic cells through the release of cytotoxic payloads in the tumor microenvironment. This activation boosts the immune response to tumors. Additionally, certain cytotoxic compounds in ADCs can cause immunogenic cell death, leading to the release of ‘danger signals’, like calreticulin, HMGB1, and ATP, which trigger dendritic cell maturation and activation. These molecules are recognized by specific cell surface receptors on dendritic cells, further enhancing the immune response. The combination of these mechanisms of action of ADC payloads ultimately leads to the cross-priming of CD8+ T-cells in the lymph nodes by activated dendritic cells. This process promotes the infiltration of CD8+ T-cells into the tumor core, where they can engage in the cytolytic killing of tumor cells through the release of granzyme B and perforin by effector T-cells. Moreover, the abundance of CD8+ T-cells has been suggested as a means of predicting the efficacy of immune checkpoint-suppression therapy [50]. Essentially, ADCs have the potential to enhance anti-tumor effectiveness by prompting CD8+ T-cells to penetrate the tumor and heighten its responsiveness to CPI treatment [51]. A notable example is the TROPHY-U-01 study, a multi-cohort, open-label, phase II trial. The first cohort of this study involved metastatic urothelial carcinoma (mUC) patients who had previously experienced treatment failure with platinum chemotherapy and CPI [22]. According to an analysis of data from various cohorts, SG (SG) demonstrated encouraging effectiveness and a consistent positive outcome in mUC patients who were either new to platinum treatment or resistant to it. Furthermore, the combination of SG and CPI could offer a new therapeutic approach for mUC patients who have not been previously treated with CPI [22,47,52]. Additionally, the BEGONIA study was specifically designed to assess the effectiveness and safety of combining durvalumab with Dato-DXd as a first-line treatment for TNBC. The preliminary data from this study indicated a favorable safety profile and a noteworthy response rate with the combination therapy. An ongoing translational data analysis is being conducted to further substantiate the efficacy and safety of this treatment approach [53].

#### 3.2.4. Combination with Other ADCs

The co-expression of multiple targets in the same cancer presents an opportunity to enhance treatment outcomes through the development of bi-specific ADCs or combination ADC therapies. For instance, in urothelial carcinoma, both NECTIN4 and Trop2/TACSTD2 are frequently overexpressed, and their expressions are closely linked. Additionally, HER2/ERBB2 is correlated with ERBB3 and Trop2/TACSTD2 [54]. The combination of ADCs targeting different receptors represents an innovative approach to cancer therapy. Synergistic effects can be achieved by concurrently targeting multiple crucial survival pathways in tumor cells to enhance treatment efficacy and mitigate the development of drug resistance. The initial results from a phase I trial combining two ADCs in the treatment of malignant tumors demonstrate that the combination of SG and EV (enfortumab vedotin) is both safe and feasible in patients with drug-resistant metastatic uroepithelial carcinoma, leading to a high early response rate [55]. Currently, a phase II clinical study (NCT06100874) is enrolling individuals to evaluate the safety and effectiveness of SG in combination with trastuzumab for metastatic HER2+ breast cancer.

#### 3.2.5. Other Potential Combination Strategies

Radiation therapy is a frequently utilized method in the treatment of cancer, activating an immune response against tumors through the induction of cancer cell death and the release of antigens from within tumor cells. Precise levels of radiation have the ability to create new antigens and boost the immune response against tumors that have a high rate of mutation [56]. Furthermore, endocrine therapy, known for its low toxicity and good tolerability, particularly benefits hormone-dependent tumors like breast, prostate, and endometrial cancers. These theoretical foundations suggest that combining ADCs with radiotherapy or endocrine therapy could be promising for cancer patients. Two ongoing clinical trials are investigating the feasibility and safety of HER2-targeted ADCs combined with radiotherapy or endocrine therapy [57,58], offering new insights into the combined use of Trop-2-targeted ADCs. In patients with metastatic castration-resistant prostate cancer (mCRPC), the expression of TACSTD is positively correlated with AR-V7, indicating that Trop-2-targeted therapy in conjunction with endocrine therapy could potentially decrease hormone resistance [59]. A clinical trial of phase II is currently in progress to evaluate the efficacy and safety of SG in patients with mCRPC who are undergoing second-generation AR-targeted therapy (NCT03725761). More combination therapy strategies are being investigated, including the use of SG in combination with hydroxychloroquine for breast cancer (NCT06328387), and SG in combination with loperamide and G-CSF to enhance the tolerance of SG in patients with unresectable locally advanced or metastatic TNBC (NCT05520723).

## 4. Limitations

Despite advancements in Trop-2-targeted ADC therapies, challenges persist. These include the variability in Trop-2 expression, the stability of and resistance to ADCs, and the lack of predictive biomarkers of efficacy.

### 4.1. The variability in Trop-2 Expression

The expression pattern of Trop-2 in malignancies is complex. Its expression levels vary significantly between different types and stages of tumors and even within different regions of the same tumor. Toda et al. found that Trop-2 expression was significantly higher in anaplastic thyroid carcinoma (ATC) undifferentiated from papillary thyroid carcinoma (PTC) compared to ATC undifferentiated from follicular thyroid carcinoma (FTC) and de novo ATC. An immunohistochemistry (IHC) evaluation was conducted using the Allred scoring system, which involved grading specimens based on their intensity score (IS) and proportion score (PS). The IS classified specimens into four grades: 0 for negative, 1 for weak, 2 for moderate, and 3 for strong. PS determined the percentage of stained cells: 0 for 0%, 1 for <1%, 2 for 1–10%, 3 for 11–33%, 4 for 34–66%, and 5 for 67–100%. The total score (TS) was calculated by summing the IS and PS, with a rating of positive given when the TS was 3 or higher. The total score of Trop-2 was notably increased in the PTC origin-type group in comparison to the FTC origin-type and de novo-type groups [60]. Using RNA-seq data, Sperger et al. analyzed 634 metastatic castration-resistant prostate cancer (mCRPC) samples from four separate cohorts to compare TACSTD2 expression levels in tumors classified as adenocarcinoma or neuroendocrine prostate cancer (NEPC). Further sub-classification of patients with adenocarcinoma into luminal or basal subtypes revealed that tumors from these patients had markedly elevated TACSTD2 expression compared to tumors from patients with NEPC [61]. Studies on colorectal cancer have shown that moderately to highly differentiated tumor tissues exhibit increased Trop-2 expression, which is positively associated with CD9. The protein levels of Trop-2 and CD9 were examined through IHC in a case series involving 177 CRC patients. High levels of Trop-2 and CD9 were detected in moderately and poorly differentiated CRC tissues, while their expression was low in well-differentiated tissues. Furthermore, a biparametric analysis of Trop-2/CD9 in CRC identified subgroups where patients with high levels of both proteins had a significantly higher risk of metastatic relapse compared to those with low levels of Trop-2 and CD9 [62]. Moretto et al. noted significant differences in Trop-2 expression in metastatic colorectal cancer samples with varying proportions, from low to high expression, closely linked to BRAF gene mutation and tumor sites. In their study, a total of 251 tumor blocks and 135 paraffin slides from the TRIBE2 study were analyzed for Trop-2 immunohistochemical assessment. The results revealed a diverse expression pattern of Trop-2, with 23% classified as high, 30% as medium, and 47% as low. Tumors with high Trop-2 expression were more likely to have BRAF mutations and be located on the right side compared to those with medium and low expression levels [63]. Izci et al. confirmed the variability of Trop-2 expression levels across different disease stages and subtypes of TNBC. The study analyzed Trop-2 expression in TNBC patients diagnosed between 2000 and 2017 using IHC on whole-slide tumor sections. The expression levels were evaluated as continuous and categorical variables, with H-scores categorized as high (201–300), medium (100–200), and low (<100). The results showed that Trop-2 expression was high in 97 (16.5%) patients, medium in 149 (25.3%) patients, and low in 343 (58.2%) patients [64]. In oral squamous cell carcinoma (OSCC), Erber et al. identified a significant correlation between high central tumoral and lower peripheral Trop-2 expression patterns and impaired Recurrence-Free Survival (RFS). Their study, using IHC, examined Trop-2 expression in both central and peripheral tumor regions, as well as non-neoplastic oral mucosa. OSCC patients with high central and low peripheral Trop-2 expression had a five-year overall survival (OS) rate of 41.2%, compared to 55.6% for those with different expression patterns. The study revealed that high central tumoral and lower peripheral Trop-2 expression were associated with reduced OS and RFS in OSCC patients, even after adjusting for other factors in the multivariate Cox regression analysis [59]. In summary, the diverse expression patterns of Trop-2 present a considerable challenge for therapeutic strategies.

### 4.2. The Stability of and Resistance to ADCs

The stability of ADCs is crucial for their efficacy and safety. To effectively target tumor cells in vivo, ADC drugs must accurately and promptly deliver cytotoxic payloads. A detailed analysis revealed that the toxicity, dose-limiting effects, and maximum tolerated dose of ADC drugs were not directly related to the level of target antigen expression, but rather to the type of linker utilized [65]. Research has shown that drug instability can cause premature dissociation in vivo, leading to nonspecific release and subsequent adverse effects [66]. Despite efforts to improve the stability of ADC linkers, treatment-related adverse reactions, especially dose-limiting hematotoxicity, are common in clinical trials. For instance, the phase I/II IMMU-132-01 basket trial demonstrated IMMU-132’s efficacy in advanced TNBC but reported significant treatment-related adverse events, like nausea, diarrhea, fatigue, alopecia, and neutropenia [67]. Similarly, the ASCENT study, a phase III clinical trial for advanced TNBC treatment, revealed that patients receiving SG experienced more adverse events than those on traditional monotherapy, including neutropenia, leukopenia, diarrhea, anemia, and febrile neutropenia [16]. A fatal adverse event related to SG involving septic shock from neutropenic colitis was reported in TROPiCS-02 [27].

Additionally, mechanisms of resistance to ADCs, including altered target antigen expression, defective internalization pathways, and lysosomal dysfunction, have been a focus of research [68]. Recent studies have highlighted the production of tumor neoantigens in response to low-dose radiation as a potential strategy to address ADC resistance [56]. Nevertheless, the non-specific effects of an off-target payload remain a significant factor contributing to various tolerance phenomena observed in clinical settings [66].

### 4.3. Lack of Predictive Biomarkers of Efficacy

The levels of estrogen and progesterone receptors, along with human epidermal growth factor receptor 2 (HER-2), have become key biomarkers in breast cancer treatment for hormone therapy and targeted HER-2 therapy. Despite the potential value of Trop-2 expression levels in affecting sensitivity to Trop-2-targeted therapy, current treatment guidelines do not require Trop-2 expression levels to be tested before the use of Trop-2-targeted ADCs for breast or uroepithelial carcinoma patients. Clinical study analyses revealed that patients with varying Trop-2 expression levels derive benefits from ADC treatment, but the high expression does not significantly correlate with improved outcomes, possibly due to the bystander effect [69,70,71,72]. Other biomarkers associated with Trop-2 expression, such as SLFN11 (a predictor of SN-38 treatment sensitivity), Rad51 (involved in homologous recombination repair), and ERCC1, can also serve as important predictors of treatment outcomes [73].

## 5. Future Direction

### 5.1. Antibody Design

Further research into the function of Trop-2 in normal and tumor cells will enhance our understanding of Trop-2’s potential and limitations as a therapeutic target. This will enable the development of more precise antibodies. Trop-2 cleavage by ADAM10 is prevalent in various tumor types, such as skin, ovarian, colon, and breast cancer, while not being observed in normal cells [74,75]. The cancer-specific antibody Hu2G10, developed based on this finding, exhibits a significantly higher affinity for cancer-specific Trop-2 compared to wtTrop-2. In vivo studies demonstrate that the naked Hu2G10 antibody effectively inhibits the growth of breast cancer, colon cancer, ovarian cancer, and prostate cancer, thereby enhancing anti-cancer efficacy and paving the way for the next generation of ADCs [76,77,78,79]. Furthermore, exploring the heterogeneity of Trop-2 is expected to lead to the development of antibodies with improved affinity. Trop-2 molecules have been found to form stable dimers and polymers at interfaces of cancer cells, potentially hindering access to antibody-binding sites in densely packed cancer cell populations. The 2EF mAb was specifically designed to overcome this challenge and has shown enhanced efficacy against various tumor models, penetrating deeper into cancer sites, while also exhibiting reduced toxicity in animal studies. Consequently, the humanized antibody Hu2EF-7 has emerged as a promising candidate for the development of novel anti-Trop-2 ADCs [80].

### 5.2. Influence of Extracellular Vesicles on ADCs

Extracellular vesicles (EVs) are small membrane structures secreted by cells that play a crucial role in maintaining cellular physiological balance and in pathological processes. They are categorized into exosomes and microvesicles and are capable of transporting proteins, lipids, RNA, and other biological molecules into the extracellular environment [81]. The exceptional target specificity, biocompatibility, low immunogenicity, and inherent stability of extracellular vesicles have positioned them as promising drug delivery systems in recent years, with significant potential in precision tumor therapy. Furthermore, extracellular vesicles released by tumor cells may carry specific biomarkers, offering a novel approach for early tumor diagnosis, prognosis assessment, and therapeutic monitoring. Moreover, these vesicles can modulate communication among tumor cells by promoting angiogenesis and modifying the extracellular matrix, thus impacting tumor growth and metastasis [82,83]. The study of extracellular vesicles in tumor therapy is currently focused on their potential and practicality as drug delivery vehicles.

Extracellular vesicles can improve drug targeting and stability [84,85,86]. Yong et al. provided theoretical validation for exosomes as effective targeted drug carriers for cancer therapy [87]. Li et al. discovered that drug-loaded extracellular vesicles with specific targeting ligands coated with high-density antibodies can enhance their ability to target tumors and effectively combat cancer [88]. Saari et al. utilized tumor cell-derived EVs to deliver the drug PTX, showcasing its enhanced targeting and cytotoxicity against prostate cancer [89]. Niu et al. created bionic EV-DNs that can surpass the limitations of the blood–brain tumor barrier, enabling efficient drug delivery and significantly enhancing anti-tumor efficacy [90], while Zhang et al. demonstrated that liposomes can effectively deliver drugs to the brain without compromising the integrity of the blood–brain barrier [91]. Feng et al. showed that folic acid assembly in extracellular vesicles can improve drug delivery, regulate the tumor microenvironment, and directly inhibit tumor metastasis, thus enhancing therapeutic outcomes [92].

Extracellular vesicles (EVs) expressing Trop-2 have the potential to enhance the effectiveness of Trop-2-targeted ADCs by delivering drugs to Trop-2-negative tumor cells or tumor stromal cells. For instance, brentuximab vedotin and trastuzumab emtansine can utilize EVs carrying CD30-positive and HER2-positive markers released by tumor cells to target neighboring tumor cells lacking these antigens, leading to tumor regression [93,94,95] (Figure 1). Prostate cancer cells release extracellular vesicles (EVs) that express Trop-2 [96]. These EVs have the potential to transfer their contents to cancer cells in the same tumor that do not express Trop-2, causing a bystander effect. Additionally, Trop-2-expressing EVs could potentially transport ADCs to other cells in the tumor microenvironment, thereby inhibiting cancer proliferation [97].

Extracellular vesicles can also influence drug resistance. Wang et al. demonstrated that extracellular vesicles derived from human gastric epithelial cells can deliver miR-214 to overcome cisplatin chemotherapy resistance in gastric cancer [98]. Liang et al. utilized engineered extracellular vesicles to deliver anti-tumor drugs (5-FU and miR-21 inhibitors), resulting in enhanced tumor cytotoxicity and the reversal of cancer cell resistance to 5-FU [99].

In conclusion, EVs offer new strategies for further optimizing ADC design. The high biocompatibility and low immunogenicity of EVs can effectively improve the distribution and release efficiency of ADCs. As promising drug delivery vehicles, EVs have great potential in improving efficacy, reducing toxicity, and overcoming drug resistance to Trop-2-targeted ADCs.

### 5.3. Multi-Target Combination Therapy

The co-expression of multiple different ADC targets within the same tumor provides an opportunity for the development of future clinical trials involving combinations of ADCs or the creation of bi-specific ADCs, for example, combining anti-NECTIN4 with anti-Trop-2 in lung, breast, and ovarian cancer or anti-HER2 with anti-ERBB3 in lung, breast, and prostate cancer [54,63]. Additionally, in solid tumors with high Trop-2 expression, various other potential therapeutic targets exhibit heterogeneity in specific tumor types. For example, there is an increase in FOXM1 expression in breast cancer, hepatocellular carcinoma, and basal cell carcinoma. TP63 and WT1 are also prominently expressed in squamous cell carcinoma, bladder cancer, breast cancer, and gastric cancer [100,101,102]. In colorectal cancer, the positive correlation between Trop-2 and CD9 expression opens up opportunities for combined targeted therapy strategies [62].

One promising future direction for ADCs is to investigate combination strategies of different target ADCs to improve drug efficacy. Optimizing combination therapy through rational administration sequences and dose combinations is essential. Furthermore, exploring ADCs with dual targets or multiple payloads is a worthwhile consideration.

### 5.4. Personalized Treatment

Tools to predict the drug sensitivity of each patient can be helpful in guiding treatment decisions. At present, there is no ideal strategy to assist clinicians in choosing the appropriate treatment for patients. While traditional tumor classification methods can help guide treatment to a certain extent, they cannot effectively identify the optimal group for Trop-2-targeted medication. The effectiveness of ADCs is closely related to their mechanism of action. The accurate identification and selection of potential responsive populations have emerged as pressing issues in the field of tumor therapy. Most of the currently approved Trop-2-targeted ADCs are inhibitors of topoisomerase 1 (TOP1), making high TOP1 expression a potential indicator of sensitivity to camptothecin analogues. Furthermore, the presence of the SN-38-sensitivity predictor SLFN11, HRR-related protein Rad51, and ERCC1 has shown promise in predicting clinical benefits [73,103]. Additionally, CTSL, which hydrolyzes T-DXd peptide ligators, is elevated in advanced breast cancer. BCRP/ABCG2, responsible for mediating camptothecin’s effects and contributing to the bystander effect observed with T-DXd, is upregulated in metastatic compared to primary breast cancer, among other types [54]. Although this field of research is still in its early stages, it is a crucial step towards achieving precision medicine. Therefore, the precise identification of populations sensitive to ADC treatment is a key direction for future exploration. Table 2 displays the relevant clinical trials.

## 6. Discussion

Precision targeted therapy, particularly in the individualized management of complex conditions like cancer, holds significant therapeutic promise. Future progress relies on advancing novel technologies, wider biomarker utilization, and a deeper understanding of underlying causes. Peptide–drug conjugates (PDCs) are the upcoming wave of targeted treatments after ADCs, providing better cell penetration and increased drug specificity [104]. PDC is a targeted therapeutic agent with a mechanism of action similar to that of ADCs, involving the conjugation of peptides and drugs to facilitate the effective delivery of chemotherapy agents to tumor cells. The three essential components of PDC include homing peptides, linkers, and cytotoxic drugs, which synergistically target receptors on tumor cells and enhance the therapeutic efficacy of peptide treatments. Currently, pharmaceutical firms are actively creating PDCs as specialized therapeutic options for ailments like cancer and metabolic disorders. PDCs demonstrate effective tumor infiltration, economical production, and a lack of immunogenicity, making them more versatile in the commercial and clinical sectors compared to other merged drugs. However, ADCs possess unique benefits over PDCs. ADCs can precisely bind to target cell surface antigens due to high affinity, enabling accurate drug delivery. They also induce strong cell killing effects through antibody-mediated cytotoxicity and complement-dependent cytotoxicity mechanisms. Furthermore, ADC technology allows toxic compounds to be stably linked to antibodies, increasing local drug concentration without systemic toxicity, enhancing therapeutic outcomes. As a whole, ADCs and PDCs are pivotal in contemporary medicine, enhancing treatment personalization and effectiveness through insights from molecular biology.

Trop-2-targeted ADCs have demonstrated substantial promise in the management of diverse solid malignancies. In the future, we look forward to improving the efficacy of Trop-2-targeted therapies by optimizing antibody design and exploring new combination therapy strategies and more personalized treatment options for cancer patients. Subsequent research is necessary to investigate how EVs can be engineered or modified to enhance their specificity and efficiency in delivering ADCs, as well as to identify more efficient biological markers of drug sensitivity.

## Figures and Tables

**Table 1:** Representative clinical trials related to Trop-2-targeted ADC combination therapy.

Combination Therapy	Identifier	Combine	Phase	Recruitment Status	Cancer Type
Chemotherapy	NCT06040970	SG + cisplatin	Phase 1/2	Recruiting	Ovarian and endometrial cancer
	NCT06065371	SG + capecitabine	Phase 1	Not yet recruiting	Gastrointestinal cancers
	NCT05089734	SG + docetaxel	Phase 3	Active, not recruiting	NSCLC *
	NCT05555732	Dato-DXd + pembrolizumab + platinum	Phase 3	Recruiting	NSCLC
Molecularly targeted drugs	NCT04039230	SG + talazoparib	Phase 1/2	Recruiting	Breast cancer
	NCT05113966	SG + talazoparib	Phase 2	Active, not recruiting	TNBC *
	NCT05143229	SG + alpelisib	Phase 1	Recruiting	Breast cancer
	NCT04826341	SG + berzosertib	Phase 1	Recruiting	Solid tumor
	NCT03992131	SG + rucaparib	Phase 1/2	Terminated	Solid tumor
	NCT05006794	SG + GS-9716	Phase 1	Recruiting	Solid tumor
	NCT05675579	Dato-DXd + osimertinib	Phase 3	Not yet recruiting	NSCLC
	NCT03944772	Dato-DXd + osimertinib	Phase 2	Not yet recruiting	NSCLC
	NCT05417594	Dato-DXd + AZD9574	Phase 1/2	Recruiting	Solid tumor
Immunotherapy	NCT05675579	SG + pembrolizumab	Phase 2	Recruiting	TNBC
	NCT06055465	SG + pembrolizumab	Phase 2	Recruiting	NSCLC
	NCT05633654	SG + pembrolizumab	Phase 3	Recruiting	TNBC
	NCT04468061	SG + pembrolizumab	Phase 2	Recruiting	TNBC
	NCT05609968	SG + pembrolizumab	Phase 3	Recruiting	NSCLC
	NCT05382286	SG + pembrolizumab	Phase 3	Recruiting	TNBC
	NCT06081244	SG + pembrolizumab	Phase 2	Recruiting	TNBC
	NCT03869190	SG + atezolizumab	Phase 1/2	Recruiting	Bladder cancer
	NCT04434040	SG + atezolizumab	Phase 2	Active, not recruiting	TNBC
	NCT03424005	SG + atezolizumab	Phase 1/2	Recruiting	Breast cancer
	NCT06133517	SG + zimberelimab (ZIM) + domvanalimab (DOM)	Phase 2	Not yet recruiting	MIBC *
	NCT05535218	SG + pembrolizumab	Phase 2	Enrolling by invitation	MIBC
	NCT06161532	SG + atezolizumab	Phase 2	Not yet recruiting	Rare genitourinary tumors
	NCT04863885	SG + ipilimumab + nivolumab	Phase 1/2	Active, not recruiting	Urothelial cancer
	NCT03337698	SG + atezolizumab	Phase 1/2	Recruiting	NSCLC
	NCT03971409	SG + avelumab	Phase 2	Recruiting	TNBC
	NCT04143711	SG + DF1001	Phase 1/2	Recruiting	Solid tumor
	NCT05633667	SG + zimberelimab (ZIM) + domvanalimab (DOM)	Phase 2	Recruiting	NSCLC
	NCT06103864	Dato-DXd + durvalumab	Phase 3	Recruiting	TNBC
	NCT05215340	Dato-DXd + pembrolizumab	Phase 3	Recruiting	NSCLC
	NCT05629585	Dato-DXd + durvalumab	Phase 3	Recruiting	TNBC
	NCT06112379	Dato-DXd + durvalumab	Phase 3	Recruiting	Breast cancer
	NCT06357533	Dato-DXd + rilvegostomig	Phase 3	Not yet recruiting	NSCLC
	NCT03742102	Dato-DXd + durvalumab	Phase 1/2	Recruiting	TNBC
	NCT06328387	SG + hydroxychloroquine	Phase 1/2	Recruiting	Breast cancer
Chemoimmunotherapy	NCT05555732	Dato-DXd + pembrolizumab + platinum	Phase 3	Recruiting	1L NSCLC
	NCT05489211	Dato-DXd + saruparib (AZD5305) + carboplatin	Phase 2	Recruiting	Solid tumors
	NCT04526691	Dato-DXd + pembrolizumab + platinum	Phase 1	Recruiting	Advanced or metastatic NSCLC
	NCT04612751	DatoDXd + durvalumab + carboplatin	Phase 1	Recruiting	Advanced or metastatic NSCLC
	NCT05687266	DatoDXd + durvalumab + carboplatin	Phase 3	Recruiting	Advanced NSCLC without AGAs *
	NCT05061550	Dato-DXd + durvalumab + platinum	Phase 2	Recruiting	NSCLC
Others	NCT06328387	SG + hydroxychloroquine	Phase 1/2	Recruiting	Breast cancer
	NCT06100874	SG + trastuzumab	Phase 2	Recruiting	Breast cancer
	NCT06244485	SG + valemetostat tosylate	Phase 1	Recruiting	Solid tumor
	NCT04724018	SG + EV	Phase 1	Active, not recruiting	Urothelial cancer
	NCT03725761	SG + enzalutamide/abiraterone/ARN-509	Phase 2	Recruiting	Prostate cancer
	NCT06238921	SG + zimberelimab with SRS *	Phase 1/2	Not yet recruiting	mTNBC * with brain metastases
	NCT05833867	SG + adaptive radiation therapy	Phase 1	Recruiting	MIBC
	NCT05520723	SG + loperamide + G-CSF	Phase 2	Active, not recruiting	TNBC
	NCT06167317	SG + GS-0201	Phase 1	Recruiting	Solid tumor

* NSCLC, non-small cell lung cancer; (m)TNBC, (metastatic) triple-negative breast cancer; MIBC, muscle-invasive bladder cancer; AGAs, actionable genomic alterations; SRS, stereotactic radiation. Data were collected up to 20 April 2024 from ClinicalTrials.gov.

**Table 2:** Representative clinical trials related to biomarkers of Trop-2-Targeted ADCs.

Identifier	Study Type	Conditions	Status
NCT06240195	Observational	TNBC	Recruiting
NCT06341478	Interventional	MIBC	Recruiting
NCT05097599	Interventional	Solid tumor	Recruiting
NCT05770531	Interventional	Breast cancer	Suspended
NCT05332561	Interventional	Breast cancer	Recruiting
NCT06311214	Interventional	Solid tumor	Not yet recruiting
NCT06178588	Interventional	Cholangiocarcinoma	Recruiting

Data were collected up to 20 April 2024 from ClinicalTrials.gov.
